# Liver metastasis of retinoblastoma

**DOI:** 10.3332/ecancer.2025.1824

**Published:** 2025-01-15

**Authors:** Elily D Apumayta, Mario Buitrago, Marco Rioja, Sandra Alarcon, Jhonatanael Salvador, Eloy Ruiz

**Affiliations:** 1 Departamento de Cirugía, Instituto Nacional de Enfermedades Neoplásicas, Surquillo 15038, Peru; 2 Departamento de Oftalmología, Instituto Nacional de Enfermedades Neoplásicas, Surquillo 15038, Peru; 3 Departamento de Patología, Instituto Nacional de Enfermedades Neoplásicas, Surquillo 15038, Peru; 4 Departamento de Oncología Pediátrica, Instituto Nacional de Enfermedades Neoplásicas, Surquillo 15038, Peru; 5 Universidad de San Martín de Porres, Santa Anita 15011, Peru; 6 Departamento de Cirugía de Abdomen, Instituto Nacional de Enfermedades Neoplásicas, Surquillo 15038, Peru; a https://orcid.org/0000-0002-1828-7009; b https://orcid.org/0009-0002-4193-3483; c https://orcid.org/0009-0008-5494-2665; d https://orcid.org/0000-0002-2399-957X; e https://orcid.org/0000-0001-5561-0752

**Keywords:** hepatic metastasis, retinoblastoma, metastasis

## Abstract

**Objective:**

To outline the clinical manifestations, imaging, management and prognosis of patients with liver metastases of retinoblastoma (RB).

**Methodology:**

Retrospective analysis of two cases diagnosed with liver metastasis of RB between 2018 and 2023 at the National Institute of Neoplastic Diseases in Lima, Peru.

**Results:**

A total of 2 (0.71%) out of 283 patients had liver metastases from RB, as confirmed by pathology. A 12-month-old female patient with unilateral RB pT1 without risk factors remains under observation after enucleation. After 5 months, she presented with multiple heterogeneous hepatic lesions up to 10 cm in size, with a hypodense center and slightly contrast-enhancing surface. She received chemotherapy and died 7 months later. The second case was a 2-year-old female with unilateral RB, pT3b and G3 with retrolaminar involvement of the optic nerve and choroidal invasion. She received adjuvant chemotherapy. After 21 months, she presented with multiple hypodense lesions with diffuse distribution in hepatic parenchyma, with distinct peripheral enhancement, some of which were confluent. She died without treatment 1 month later.

**Conclusion:**

Hepatic metastasis of RB is rare. In these two cases, they were presented as heterogeneous, predominantly hypodense lesions with mild contrast enhancement on the CT scan. These events were simultaneously associated with recurrence in the central nervous system, even in the absence of risk factors for metastasis and dissemination.

## Introduction

Although retinoblastoma (RB) is considered a rare childhood cancer, it is the most common childhood ocular neoplasm and occurs in 1 in 15,000 to 20,000 live newborns. RB represents 2% of all pediatric cancers [1–3]. Between 25% and 40% of cases are hereditary by autosomal dominant inheritance [1]. The typical age of presentation is before 5 years old, predominantly 15 months old. RB has no gender or racial preference [4, 5]. Tumours are classified into risk groups International Classification of Retinoblastoma (IRC) according to the size of tumour, localisation, spread and functional compromise [6]. Early detection and management leads to a 5-year survival rate greater than 90% [7].

Metastases occur in 4.8%–15% of patients, and they are related to factors of the primary tumour such as involvement of the optic nerve beyond the lamina cribrosa, ciliary body, choroid and deep sclera [1, 8]. Even though the RB metastasis patterns are relatively unknown, it seems that the main route of metastasis is direct infiltration to the central nervous system (CNS) through the optic nerve and the hematogenous route to the bone and bone marrow [6–9]. Liver metastasis is less frequent, but the exact rates can vary depending on the study and patient population. It is reported in less than 1% of cases [10].

In Peru, a peak incidence had been found at 2.5 years of age, predominantly in males (58%). Less than 3% of cases had a positive family history for RB. One in four cases had bilateral involvement. Approximately 50% of RB presented stage III–IV at diagnosis, which affected the prognosis. Thus, overall survival at 5 years was 100% for CD I, 99% for CD II and 82% for CD III. Finally, no patient with CD IV reached 5 years of survival [5].

Our institution, Instituto Nacional de Enfermedades Neoplásicas (INEN), which is the main cancer referral center in the country, treats 80% of RB cases nationwide. Between July 2018 and June 2023, we treated 283 patients with a diagnosis of RB. Of all patients, only 2 had liver involvement (0.70%) as disease recurrence, confirmed by pathology. This report details the clinical characteristics, management and prognosis of those patients with liver metastases from RB.

### Case 1

**Figure 1.**
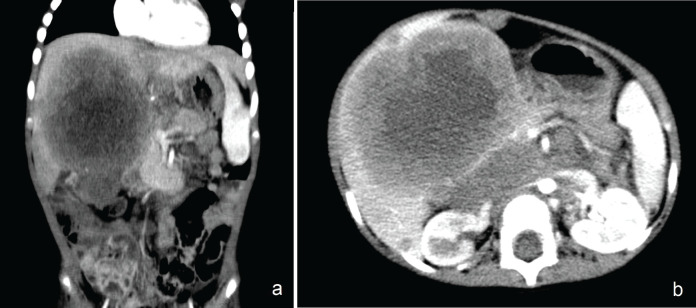
(a) Heterogeneous lesions with central hypodense areas, in hepatic segments 4b, 5, 6 and 8, the largest of 10 cm, whose surface is slightly enhanced by contrast. (b): Retroperitoneal ganglionic conglomerate surrounding the emergence of the celiac trunk, superior mesenteric artery and kidney.

A 12-month-old female, without a family history of RB, presented a heterogeneous tumour of 2 × 1.5 cm, occupying 90% of the vitreous cavity, confined to the right eyeball, with intraocular calcifications, group E of the IRC, treated with enucleation. The eye was healthy in the fundoscopic exam. The tumour was RB pT1, unifocal, G2 and invaded the optic nerve in front of the lamina cribrosa, without choroidal or extraocular involvement. Cerebrospinal fluid cytology and brain magnetic resonance imaging (MRI) were negative. Therefore, the patient did not receive adjuvant treatment and remained under observation. After 5 months, there was evidence of a painful mass in the right hypochondrium, seen by tomography as diffuse lesions in hepatic segments 5, 6 and 8, the largest being 10 cm, with a retroperitoneal ganglionic conglomerate above the renal hilum of 7.5 × 3 cm (Figure 1). Biopsy confirms RB metastases. She received three cycles of Vincristine, Doxorubicin and Cyclophosphamide chemotherapy with a partial response. She then continued with a second line of chemotherapy with vincristine, cisplatin, cyclophosphamide and etoposide. After the third course, she developed hypoactivity and vomiting. New nodular lesions of up to 9 mm in the leptomeningeal region of both cerebellar hemispheres were observed, leading to hydrocephalus. She died before receiving holocranial radiotherapy due to progression within the CNS, 7 months after the hepatic recurrence was identified.

### Case 2

**Figure 2.**
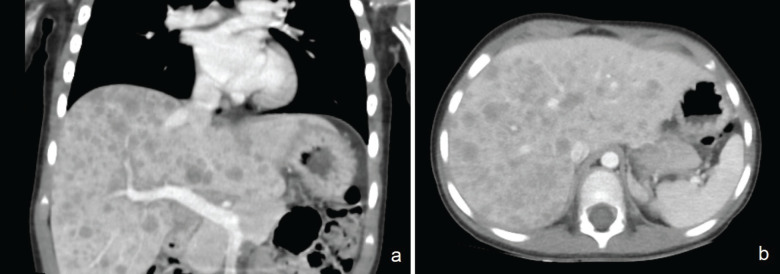
Multiple hypodense lesions of diffuse distribution in all hepatic segments, confluent, with discrete peripheral enhancement, up to 1.8 cm. (a): Coronal section and (b): axial section.

A 2-year-old female, without a family history of RB, with a 2-cm tumour in her left eye occupying 90% of the vitreous cavity with internal calcifications and conjunctival hyperemia, group E of IRC, treated with enucleation. Unifocal RB, G3 and pT3b involving the optic nerve beyond the lamina cribrosa and total choroidal invasion. Cerebrospinal fluid cytology and brain MRI were negative for the disease. She received adjuvant chemotherapy (6 courses of Vincristine, Carboplatin and Etoposide). After 21 months since the end of treatment, headache, abdominal pain and vomiting had appeared. The computed tomography (CT) scan revealed irregular tissue at the level of the sphenoid sinuses, involving posterior ethmoidal cells and extending to the cavernous sinuses. Also, multiple metastatic lesions were observed throughout the liver parenchyma (Figure 2), with histopathological confirmation of liver metastasis due to RB. She died 1 month later.

## Discussion

We present two patients up to 2 years old with a late diagnosis of unilateral RB, group E, without genetic study. The first one had a pT1 localised disease and the second had pT3b disease with high-risk histopathological features. Both patients underwent clinical staging that included fundus, ocular ultrasound under general anesthesia, cerebrospinal fluid cytology and magnetic resonance imaging of the orbit and brain. The extraocular disease was eliminated at diagnosis. They were treated with a primary enucleation of the eyeball, with no previous therapy. Both registered free margins and placement of a porous polyethylene orbital implant.

Adjuvant chemotherapy was decided according to the institutional management protocol number 9,627 and 9,628 in force since 2007, which were based on international guidelines, depending on the presence of high-risk features [11]. In this way, patient case number 2 received adjuvance. During routine follow-up, quarterly during the first year, patients underwent fundoscopic examination, hemogram and physical examination to check for cervical lymph nodes. Regular MRI scans of the brain and orbits for early detection of CNS metastasis. Bone scans and bone marrow aspirates to detect bone and bone marrow involvement. Although CT scans of the chest and abdomen are not part of routine examinations, they can help detect other visceral metastases, such as lung and liver. Each patient developed liver metastases after 5 and 21 months of age, with evidence of controlled disease after completion of treatment according to the stage. No liver dysfunction was observed.

Both patients had multiple, diffuse, rapid-growing liver metastases. One unilobar had the highest size of 10 cm, and the other had a diameter of up to 1.8 cm with bilobar distribution. Regarding imaging characteristics, no specific pattern was observed by tomography. Diffuse or single, round lesions have been described, with defined borders, low density and heterogeneity, that do not enhance to contrast, with internal calcifications and necrosis [10]. Our patients are great examples of the diverse characteristics that metastatic lesions can adopt in the liver, which can simulate microabscesses or a second neoplasm (Figures 1 and 2).

In both patients, liver metastasis was found in conjunction with metastasis to the CNS, which is a finding similar to that reported in a Chinese series. They describe five patients with liver metastasis due to RB in the context of advanced disease with the involvement of other organs and systems, without the liver being the only organ or the most likely to be involved [10]. Of the five cases reported, two died within the first and third month of diagnosis of liver metastasis. One patient died of infection secondary to chemotherapy-induced myelosuppression after disease remission, and the other patient died of multiorgan failure due to disease progression. During a mean follow-up of 9 months, two of the remaining three had partial remission and one had disease progression [10].

The most common site of distant metastasis in RB is CNS (30%–40% of metastasis cases), resulting in poor prognosis for the patient [12, 13]. The second most frequent is bone, affecting the skull and long bones mainly. In addition, bone marrow can be affected, alongside bone metastasis. Lymph nodes are usually compromised in the pre-auricular, submandibular or cervical areas, commonly due to extraocular RB. Visceral metastases, such as those in the liver, pancreas and spleen, are less frequent and often occur in conjunction with other metastatic sites [14, 15].

**Figure 3.**
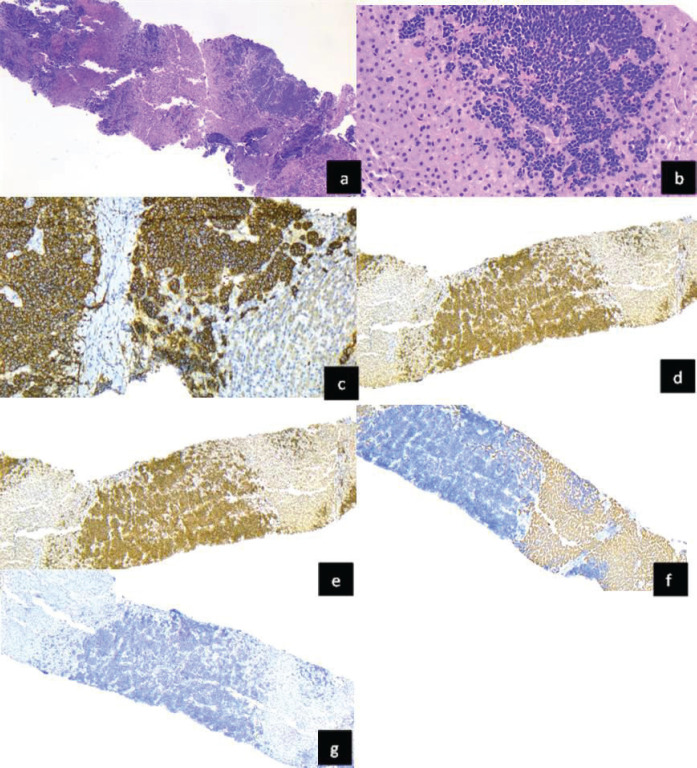
Liver tissue infiltrated by RB (a and b): Hematoxylin and eosin staining shows liver tissue infiltrated by a malignant round cell neoplasm. Immunohistochemical staining for synaptophysin positive, (c): INI -1: intact nuclear expression, (d): TdT negative, (e): Hepar 1 negative, (f): and desmin negative and (g): demonstrated hepatic metastasis of RB.

Immunohistochemistry was used to confirm liver metastasis by RB. Biopsies showed nests of tumour cells seen as solid formations extending between the liver tissue, consisting of small round blue cells with abundant chromatin (Figure 3). These were positive for synaptophysin. Although FISH was not performed to rule out other specific neoplasms through RNA sequencing, terminal deoxynucleotidyl transferase or Tdt, desmin and hepar1 were negative and helped to exclude lymphoma or leukemia, rhabdomyosarcoma and hepatocarcinoma, respectively. Considering the history of RB enucleation, liver metastasis was considered as the known primary tumour.

The management of RB depends on tumour size, location and stage at diagnosis. Small tumours up to 4 mm basal diameter and 2 mm thick can be treated with cryotherapy or photocoagulation, depending on their peripheral or posterior location, respectively. While tumours with four times the previous dimensions would benefit from brachytherapy [1, 16]. The use of enucleation is reserved for advanced tumours, with or without adjuvant therapy, depending on the presence of high-risk factors. Both patients with extensive group E tumours, according to the ICR, were treated with enucleation of the eyeball.

The definition of high-risk RB is not uniform worldwide. The 3 widely accepted high-risk factors for disseminated occult disease are an extensive RB with extraocular extension, optic nerve or choroidal invasion [17, 18]. Specifically, retrolaminar optic nerve invasion with concomitant massive choroidal or scleral invasion [17]. Some clinical prognostic factors are old age, late diagnosis, hyphema, pseudohypopyon, staphyloma, orbital cellulitis, glaucoma and buphthalmos. Another histopathological risk factor is amicroscopic residual disease after surgery. However, the disadvantage of this approach is that it is performed in the operative part of the enucleation [18].

The first patient with a pT1 tumour with preliminary optic nerve involvement, without choroidal invasion, with 40% necrosis and apoptosis. Although extensive necrosis is statistically more likely to be associated with high-risk histopathologic features, such as retrolaminar optic nerve and choroid invasion [19]. The patient did not receive adjuvant therapy because her pathological findings were not considered high-risk factors for metastasis. However, she developed liver and lymph node metastases during the course of the disease.

Among the high-risk factors described in the literature is massive choroidal invasion, greater than 3 mm in any dimension, which confers a 6% risk of extraocular disease and a lower probability of event-free survival [16]. Therefore, case 2, which presented with a pT3b, G3 tumour with total involvement of the choroid and retrolaminar involvement of the optic nerve, received adjuvant chemotherapy.

Regarding histopathological factors, the involvement of the optic nerve pre-lamina cribrosa or prelaminar, corresponds to a high-risk factor for metastasis as long as it is associated with focal choroidal invasion [16]. Other clinical high-risk factors have been previously described, which indicate advanced disease with high-risk pathology [20]. These findings were not observed in patient case 1.

The current treatment approaches for RB attempt to preserve the organ. Direct therapies, such as ophthalmic artery chemosurgery and intravitreous chemotherapy, have been replacing the use of eye enucleation, external beam radiotherapy, its adverse events and long term toxicity. However, eyes that are refractory to treatment or relapse require enucleation. Novel treatments for RB include immunotherapy, such as GD2 ganglioside and oncolytic adenovirus targeting the RB1 pathway [13]. Current treatments for advanced or recurrent diseases are currently being developed. The use of a chemoplaque, an implanted device on the surface of the eye that releases chemotherapy over a period of time, seems promising [12]. Nevertheless, systemic chemotherapy was the unique treatment option available.

Among the main causes of death in patients with RB, the development of a second malignant neoplasm, such as the pinealoblastoma and metastasis are also identified. Our two patients died of the latter within the first year of diagnosis. The first, 7 months after with a partial response to chemotherapy; and the other after 1 month without treatment. In relation to this, metastasis, especially in the CNS, increases the risk of prognosis. To counteract this, modern treatment protocols have been shown to decrease the rate of metastasis. A limitation of this report is that no molecular study of the primary tumour or metastasis was conducted in either case. It would be interesting to explore the biology of this type of metastasis. Therefore, current treatment modalities, such as targeted therapies, may improve outcomes for these patients with metastatic disease.

## Conclusion

RB exhibits diverse biological behaviour, with metastases developing in the absence of established risk factors. Strict follow-up of signs is essential for the early detection of metastases. Liver metastasis in RB is generally considered a sign of advanced disease, does not usually occur in isolation and is associated with poor prognosis. Because their CT features do not have a specific pattern, biopsy and immunohistochemistry are essential to confirm the diagnosis.

## Conflicts of interest

The authors declare that they have no conflicts of interest.

## Funding

The authors declare that they have not received any funding for this research.
